# Weekly pulmonary delivery of β-glucan-chitosan-poly(lactic co-glycolic) acid (β-C-P) nanoparticles with daily standard oral therapy achieves control of *Mycobacterium tuberculosis* in BALB/c mice

**DOI:** 10.1128/aac.00281-26

**Published:** 2026-04-20

**Authors:** Hilliard L. Kutscher, Maria Tamblin, Patrick Kenney, Jessica L. Reynolds

**Affiliations:** 1 Division of Allergy, Immunology, and Rheumatology Department of Medicine, Clinical Translational Research Center, The State University of New York at Buffalo 12292 , Buffalo, New York, USA; 2 Institute for Lasers, Photonics and Biophotonics, The State University of New York at Buffalo 12292 , Buffalo, New York, USA; 3 Department of Anesthesiology, The State University of New York at Buffalo 12292 , Buffalo, New York, USA; 4 Adult and Pediatric Infectious Disease, The State University of New York at Buffalo 12292 , Buffalo, New York, USA; Queen Mary University of London, London, United Kingdom

**Keywords:** tuberculosis, *Mycobacterium tuberculosis*, rifampin, pyrazinamide, isoniazid, nanoparticles, PLGA, β-glucan

## Abstract

Prolonged daily tuberculosis therapy contributes to toxicity, poor adherence, and drug resistance. We developed rifampin (RIF)-loaded β-glucan-chitosan-poly (lactic co-glycolic) acid nanoparticles (β-C-P nanoparticles) for weekly pulmonary delivery and evaluated a hybrid dosing regimen in a BALB/c *Mycobacterium tuberculosis* model. Weekly instilled RIF loaded 20% β-C-P nanoparticles combined with daily oral isoniazid (INH) and pyrazinamide (PZA) reduced lung and spleen bacterial burdens comparable to oral daily standard of care (RIF, INH, PZA) and were well tolerated, demonstrating preserved efficacy with reduced RIF dosing frequency.

## INTRODUCTION

Tuberculosis (TB) caused by *Mycobacterium tuberculosis* (*Mtb*) remains a major global health threat responsible for over 1.5 million deaths annually (1, 2). First-line therapy includes rifampin (RIF), isoniazid (INH), and pyrazinamide (PZA), which require prolonged daily administration (3). This extended treatment duration, combined with drug-associated toxicities, often results in poor patient compliance, treatment failure, and the development of multidrug-resistant tuberculosis (MDR-TB) (4, 5). Despite being introduced more than five decades ago, these agents remain the foundation of TB treatment, with only recently introduced second-line therapeutics, bedaquiline, pretomanid, and linezolid available to address MDR-TB (6). These limitations underscore the need for strategies that reduce dosing burden while maintaining efficacy.

Nanoparticle-based drug delivery systems, including poly(lactic-co-glycolic) acid (PLGA) nanoparticles, offer a promising approach by enabling sustained drug release and enhanced macrophage targeting, a primary cellular target of *Mtb*. Incorporation of immune-stimulatory polysaccharides, such as β-glucans, may further enhance macrophage uptake while permitting controlled immune activation (7–10). Building on these principles, we developed β-glucan-chitosan PLGA (β-C-P) nanoparticles loaded with RIF (8, 9, 11, 12). Our prior studies have demonstrated targeted uptake of this RIF-loaded β-C-P nanoparticle by alveolar macrophages (8, 9, 11–13). We previously reported short-term (7 days) (9) and long-term (28 days) (13) pharmacokinetic studies that demonstrated weekly oropharyngeal aspiration (OPA) administration of RIF loaded β-C-P nanoparticles achieved higher pulmonary RIF concentrations compared to orally administered unformulated RIF and sustained drug levels within alveolar macrophage. Furthermore, weekly instillation of RIF-loaded β-C-P nanoparticle reduced bacterial burden by 0.5–1.1 log_10_ in a low-dose BALB/c mouse TB model (13). Building on these prior studies of instilled RIF-loaded β-C-P nanoparticle monotherapy, the current study evaluates the performance of the identical RIF-loaded β-C-P nanoparticle formulation and dosing routes within a multidrug standard of care regimen, reflecting the clinical context in which RIF is used. Our previously published sustained RIF exposure profiles support the rationale that intermittent dosing of this RIF-loaded β-C-P nanoparticle can preserve antimicrobial efficacy despite reduced dosing frequency. Accordingly, this study tests whether a pharmacodynamically informed, reduced-frequency dosing strategy can maintain efficacy when incorporated into combination therapy. Specifically, we evaluated whether combining weekly instilled RIF loaded 20% β-C-P nanoparticles with daily oral gavage of INH and PZA (standard of care without RIF) could achieve efficacy comparable to daily oral gavage of RIF, INH, and PZA (standard of care) (Table 1) in a low-dose BALB/c mouse TB model.

**TABLE 1:** Description of treatment groups^*a*^

Treatment groups	% Nanoparticle Solids (w/v)	RIF (mg/kg)	INH (mg/kg)	PZA (mg/kg)	Dose route	Dose frequency
Control (no treatment)	N/A	N/A	N/A	N/A	N/A	N/A
Standard of care (RIF, INH, PZA)	N/A	10	25	150	Gavage	Daily (5/7)
Standard of care (−RIF) + 20% β-C-P nanoparticles	N/A	N/A	25	150	Gavage	Daily (5/7)
	20%	4.33	N/A	N/A	OPA	Weekly
20% β-C-P nanoparticles	20%	4.33	N/A	N/A	OPA	Weekly

^*a*^ Note: 20% solids = 200 mg/mL PLGA = 10 mg PLGA/50 µL dose = 333 mg PLGA/kg body weight = 4.33 mg RIF/kg. The reduced RIF exposure of 4.33 mg/kg is a result of the 20% β-C-P being maximally loaded. RIF, rifampin; INH, isoniazid; PZA, pyrazinamide; OPA, oropharyngeal aspiration, N/A = not applicable.

## RESULTS AND DISCUSSION

We have previously demonstrated that weekly instilled RIF loaded 20% β-C-P nanoparticles, when directly compared with daily oral RIF administration, resulted in a statistically significant reduction in pulmonary bacterial burden. Compared to untreated controls, this reduction was greater in the RIF-loaded 20% β-C-P nanoparticle group (1.12 log_10_-fold reduction) than in the daily oral RIF group (1.08 log_10_-fold reduction), indicating comparable efficacy of the instilled nanoparticle formulation in a low dose BALB/c mouse TB model (13). Building on these findings, we next evaluated the performance of the instilled RIF-loaded β-C-P nanoparticles within treatment regimens that more closely reflect standard of care therapy. Relative to untreated controls, treatment with RIF loaded 20% β-C-P nanoparticles alone administered via OPA resulted in a significant 1.12 log_10_-fold reduction in lung burden. Daily oral standard of care (RIF, INH, PZA) produced a significant 1.55 log_10_-fold reduction compared to control. The hybrid combination of RIF-loaded 20% β-C-P nanoparticles administered via OPA with daily oral INH and PZA (standard of care −RIF) led to a significant 1.50 log_10_-fold reduction in lung burden after 4 weeks of treatment (Table 2). There was no statistically significant difference between daily oral standard of care (RIF, INH, PZA) and the hybrid combination of weekly instilled RIF loaded 20% β-C-P nanoparticles with daily oral INH and PZA (standard of care −RIF), demonstrating that weekly pulmonary delivery of RIF via 20% β-C-P nanoparticles can preserve combination therapy efficacy despite reduced dosing frequency. In the spleen, relative to untreated controls, standard of care treatment (RIF, INH, PZA) resulted in a 3.27 log_10_-fold reduction; the hybrid combination of daily oral INH and PZA (Standard of care −RIF) with RIF loaded 20% β-C-P nanoparticles produced a 3.20 log_10_-fold reduction; and treatment with RIF loaded 20% β-C-P nanoparticles alone led to a 1.19 log_10_-fold reduction in bacterial burden after up to 4 weeks of treatment. Combined, these findings in the lung and spleen are consistent with prior work demonstrating effective reduction of bacterial burden following weekly dosing (13). Additionally, all treatment groups maintained or gained body weight throughout the study (Fig. S1), and no behavioral or clinical signs of distress were observed, indicating that substitution of daily oral RIF with weekly instilled β-C-P nanoparticles did not compromise tolerability.

**TABLE 2:** CFU counts in the lung and spleen^*a*^

Treatment groups	Lung	Spleen
Control	6.97 ± 0.04	6.57 ± 0.07
Standard of care	4.49 ± 0.12****	2.01 ± 0.11****
Standard of care (−RIF) + 20% β-C-P nanoparticles	4.65 ± 0.05****	2.05 ± 0.08****
20% β-C-P nanoparticles	6.24 ± 0.14***	5.52 ± 0.08****

^*a*^ BALB/c mice were exposed to a low-dose aerosol infection. Treatments were initiated on Day 28 post-infection, designated as Treatment Day 0 (T0). Mice received once weekly doses of 20% β-C-P nanoparticles (T0, T7, T14, and T21) via OPA. Standard of care therapy or standard of care (−RIF) was administered by oral gavage 5 out of 7 days. After 4 weeks of treatment, mice were euthanized on T28. Statistical analysis was performed by one-way ANOVA, followed by Tukey’s multiple comparisons test. Data are compared to control. Data shown represent the mean of lung log_10_ CFU or spleen log_10_ CFU ± SEM. *** = *P* ≤ 0.001; **** = *P* ≤ 0.0001. OPA, oropharyngeal aspiration; CFU, colony-forming units.

The present results are consistent with prior reports demonstrating that delivery of drug-loaded PLGA or lipid-based formulations can achieve bacterial reductions comparable to daily oral dosing (14, 15). Among these platforms, PLGA-based nanomaterials are one of the most extensively studied and translationally advanced approaches for TB drug delivery (16–19); notably, orally administered PLGA nanoparticles have progressed to clinical evaluation and shown increased systemic RIF exposure (15, 20–24), establishing PLGA as a clinically viable carrier system. While prior studies have investigated RIF-based nanoparticle formulations, these efforts have generally evaluated the nanoparticle either as a standalone intervention or within simplified treatment regimens (23, 25–27). In contrast, our study is among the first to systematically evaluate a weekly instilled RIF-loaded 20% β-C-P nanoparticle in combination with daily oral INH and PZA. This design more closely reflects clinically relevant TB therapy and informs how β-C-P nanoparticles perform within a multidrug treatment framework. Thus, the incremental advance provided by this brief communication builds upon a larger body of work and demonstrates how RIF-loaded 20% β-C-P nanoparticles could integrate with, and potentially enhance, standard multidrug therapy rather than replace it. The preserved efficacy observed here is consistent with sustained pulmonary drug exposure, which may compensate for reduced dosing frequency without disrupting established combination therapy paradigms.

Beyond demonstrating preserved short-term efficacy, the present results provide insight into how reduced-frequency, instilled RIF delivery by β-C-P nanoparticles may be integrated into existing combination TB therapy. Many prior nanoparticle-based TB studies have focused on either monotherapy approaches or full reformulation of multidrugs (23, 25–27). While these strategies have demonstrated proof-of-concept efficacy, they often deviate substantially from clinical treatment paradigms. In contrast, the hybrid regimen evaluated here preserves the structure of standard of care by reformulating only RIF while maintaining daily oral administration of INH and PZA. This approach minimizes disruption to established regimens and may simplify translational development relative to fully nanoparticle-based multidrug systems. RIF represents a particularly suitable candidate for reduced-frequency pulmonary delivery given its central role in TB treatment, its dose-limiting systemic toxicities, and its propensity for drug-drug interactions (28, 29). Sustained pulmonary exposure achieved through instilled β-C-P nanoparticles may reduce systemic exposure, as supported by prior pharmacokinetic observations (13).

While these results demonstrate short-term efficacy in a low-dose BALB/c model, limitations remain. This model does not fully recapitulate the heterogeneous lesion pathology of human TB, and longer treatment durations will be required to assess sterilizing activity and relapse prevention. Nonetheless, these findings support further evaluation of β-C-P nanoparticles in more pathologically complex models and provide a strong rationale for continued development of instilled, reduced-frequency RIF delivery strategies within combination TB therapy.

## SUPPLEMENTAL MATERIAL

Supplemental material aac.00281-26-s0001.docx Supplemental methods; Fig. S1 and S2.

ASM does not own the copyrights to Supplemental Material that may be linked to, or accessed through, an article. The authors have granted ASM a non-exclusive, world-wide license to publish the Supplemental Material files. Please contact the corresponding author directly for reuse.
