# Supplementary material for: Weekly pulmonary delivery of β-glucan-chitosan-poly(lactic co-glycolic) acid (β-C-P) nanoparticles with daily standard oral therapy achieves control of Mycobacterium tuberculosis in BALB/c mice
Source: Antimicrob Agents Chemother. 2026 Apr 20;70(6):e00281-26. doi: 10.1128/aac.00281-26 (PMC13231870; doi:10.1128/aac.00281-26)
Supplement: Supplemental material — Supplemental methods; Fig. S1 and S2. [file aac.00281-26-s0001.docx]

**Supplement**

**Methods**

**β-C-P nanoparticle synthesis**

RIF-loaded β-glucan-CS-PLGA (β-C-P) nanoparticles were prepared *via* a water/oil/water emulsion, followed by solvent evaporation and characterization as previously described [9, 13]. The RIF loaded 20% β-C-P nanoparticles were ~246 nm in diameter (see Supplemental Figure 2) with 6.5 ± 0.95% drug loading as previously described [9, 13].

**Oral standard-of-care dose preparation**

RIF stock solution - Rifampin was dissolved in Kollisolv® (BASF) to a stock concentration of 100 mg/ml.

INH stock solution - INH was dissolved in PBS to a stock concentration of 16 mg/ml.

PZA stock solution - PZA was dissolved in 150 mg/ml β-cyclodextrin solution heated to 60°C to a stock concentration of 18 mg/ml.

Standard of Care (RIF, INH, PZA): The resulting stock solutions were combined to create a final solution of 1 mg/ml RIF, 15 mg/ml PZA, 2.5 mg/ml INH, 125 mg/ml β-cyclodextrin, and 1% Kollisolv in PBS. Standard of Care (-RIF) + 20% β-C-P nanoparticles: The resulting stock solutions were combined to create a final solution of 15 mg/ml PZA, 2.5 mg/ml INH, 1% Kollisolv, 125 mg/ml β-cyclodextrin in PBS.

***In vivo Mtb* exposure and dosing**

All procedures involving animals were reviewed and approved by the Institutional Animal Care and Use Committee of the University at Buffalo (IACUC number: PROTO202300005). Eight-week-old, female BALB/c mice (Envigo, Indianapolis, IN) were exposed to a low-dose aerosol infection (Strain Erdman K01 [TMC 107] NR-15404; BEI Resources) using a AERO3G Whole Body Inhalation System (Biaera Technologies, Hagerstown, MD) resulting in an average of 50 to 100 *Mtb* bacilli into the lungs of each mouse [13, 30-35]. Treatments were initiated on Day 28 post-infection, designated Treatment Day 0 (T0) as previously described [13]. Mice received a once weekly dose of RIF loaded 20% β-C-P nanoparticles (T0, T7, T14, and T21) via OPA, in which isoflurane-anesthetized animals were administered a 50 µL bolus of RIF-loaded 20% β-C-P nanoparticles to the back of the throat and instilled the liquid, resulting in pulmonary delivery [9, 13, 36]. Standard of care was administered by oral gavage 5 out of 7 days as a 10 µL/g bolus dose using a 20-gauge feeding tube (Instech, Plymouth Meeting, PA [9, 13]. After four weeks of treatment, mice were euthanized on T28. Whole lungs and spleen from infected mice were aseptically removed, weighed, and homogenized in ice-cold PBS using a Precellys Evolution homogenizer (Bertin Corporation, Rockville, MD). Serial dilutions of the homogenate were plated on 7H11 agar [13, 32, 35, 37-39]. Viable bacterial counts were calculated for each organ and expressed as log_10_ CFU. Analysis was done by a one-way ANOVA (analysis of variance) followed by Tukey’s correction for multiple comparisons test. All analyses were performed using Prism, (v. 10.4; GraphPad Software, San Diego, CA).


**REFERENCES**

30. Kramnik, I. and G. Beamer, *Mouse models of human TB pathology: roles in the analysis of necrosis and the development of host-directed therapies.* Semin Immunopathol, 2016. **38**(2): p. 221-37.

31. Robertson, G.T., et al., *Comparative Analysis of Pharmacodynamics in the C3HeB/FeJ Mouse Tuberculosis Model for DprE1 Inhibitors TBA-7371, PBTZ169, and OPC-167832.* Antimicrob Agents Chemother, 2021. **65**(11): p. e0058321.

32. Irwin, S.M., et al., *Presence of multiple lesion types with vastly different microenvironments in C3HeB/FeJ mice following aerosol infection with Mycobacterium tuberculosis.* Dis Model Mech, 2015. **8**(6): p. 591-602.

33. Walter, N.D., et al., *Lung microenvironments harbor Mycobacterium tuberculosis phenotypes with distinct treatment responses.* Antimicrob Agents Chemother, 2023. **67**(9): p. e0028423.

34. Lanoix, J.P., A.J. Lenaerts, and E.L. Nuermberger, *Heterogeneous disease progression and treatment response in a C3HeB/FeJ mouse model of tuberculosis.* Dis Model Mech, 2015. **8**(6): p. 603-10.

35. Driver, E.R., et al., *Evaluation of a mouse model of necrotic granuloma formation using C3HeB/FeJ mice for testing of drugs against Mycobacterium tuberculosis.* Antimicrob Agents Chemother, 2012. **56**(6): p. 3181-95.

36. Alluri, R., et al., *Open Tracheostomy Gastric Acid Aspiration Murine Model of Acute Lung Injury Results in Maximal Acute Nonlethal Lung Injury.* J Vis Exp, 2017(120).

37. Gonzalez-Juarrero, M., et al., *Mouse model for efficacy testing of antituberculosis agents via intrapulmonary delivery.* Antimicrob Agents Chemother, 2012. **56**(7): p. 3957-9.

38. Gonzalez-Juarrero, M., et al., *Preclinical Evaluation of Inhalational Spectinamide-1599 Therapy against Tuberculosis.* ACS Infect Dis, 2021. **7**(10): p. 2850-2863.

39. Gonzalez-Juarrero, M., et al., *Immune response to Mycobacterium tuberculosis and identification of molecular markers of disease.* Am J Respir Cell Mol Biol, 2009. **40**(4): p. 398-409.

**Supplemental Figure 1.** Percent Change in Body Weight of BALB/c Mice. There was no statistical significance in the change in percent body weight compared to control for T0, 7, 14, 21, and 28.


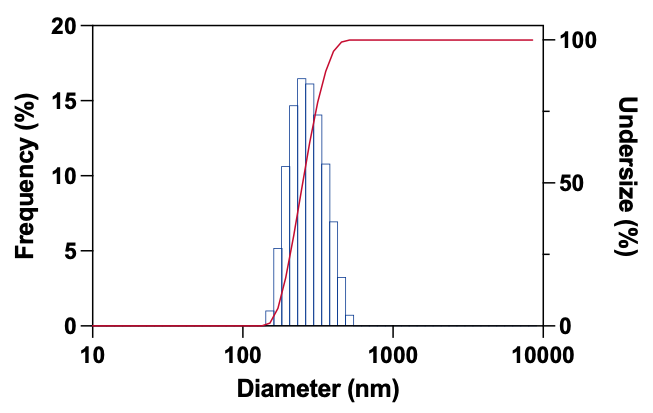


**Supplemental Figure 2.** Representative dynamic light scattering (DLS) histogram. The z-average size is 246 nm with a PDI of 0.263.
